# A risk assessment model for chronic ankle instability: indications for early surgical treatment? An observational prospective cohort – study protocol

**DOI:** 10.1186/s12891-018-2124-5

**Published:** 2018-07-18

**Authors:** Gwendolyn Vuurberg, Lauren M. Wink, Leendert Blankevoort, Daniel Haverkamp, Robert Hemke, Sjoerd Jens, Inger N. Sierevelt, Mario Maas, Gino M. M. J. Kerkhoffs

**Affiliations:** 1 Orthopaedic Research Center Amsterdam, Department of Orthopedic Surgery, Amsterdam Movement Sciences, Academic Medical Center, University of Amsterdam, Meibergdreef 9, 1105 AZ Amsterdam, The Netherlands; 2 Academic Center for Evidence based Sports medicine (ACES), Amsterdam, The Netherlands; 3 Amsterdam Collaboration for Health and Safety in Sports (ACHSS), Amsterdam, The Netherlands; 4 Department of Musculoskeletal Radiology, Amsterdam Movement Sciences, Academic Medical Center, University of Amsterdam, Meibergdreef 9, 1105 AZ Amsterdam, The Netherlands; 5 Amsterdam Movement Sciences, VU Medical Center, Department of Surgery, De Boelelaan 1117, 1081 HV Amsterdam, The Netherlands; 6 Slotervaart Medical Center, Department of Orthopedic Surgery, Louwesweg 6, 1066 EC Amsterdam, The Netherlands; 7 Slotervaart Center of Orthopedic Research & Education (SCORE), Amsterdam, The Netherlands

**Keywords:** Chronic instability, Ankle sprain, Prognosis, Ankle geometry, Model methodology

## Abstract

**Background:**

Chronic ankle instability (CAI) is a common result of an ankle sprain. Even though early surgical treatment yields the best results, overall only professional athletes are eligible for acute surgical stabilization. Treating all patients with early surgical stabilization leads to a high amount of unnecessary invasive interventions, as not all patients progress to CAI. If patients at risk of developing CAI can be identified, treatment policies may be applied more effectively and efficiently. The purpose of this study is to develop a risk assessment model to identify patients at risk for CAI that should receive early surgical treatment.

**Methods:**

In this observational prospective cohort, all patients aged sixteen years and older, reporting at the emergency department of one of the participating hospitals after sustaining a lateral ankle sprain, and filled out 1 out of 3 follow-up questionnaires and the 1 year follow-up are included. A lateral and anteroposterior radiograph is made. Patients are excluded if a fracture or other pathology is present. The included patients receive four questionnaires, including questions focusing on the sprain, treatment and complaints, the Foot and Ankle Outcome Score and the Cumberland Ankle Instability Tool. A total of eleven radiographic variables are assessed for inter- and intra-observer reliability. Additionally, four factors extracted from the questionnaires, will be evaluated for correlation with CAI. Significantly correlating factors (e.a. risk factors) will be implemented in a risk assessment model. For the final model, based on sixteen variables with a minimum of 20 events per variable and a prevalence of 30–40% after an initial sprain, a sample size of 2370 patients is needed to perform both internal and external model validation.

**Discussion:**

This study will develop the first large scale model for the risk at CAI after an ankle sprain combining radiographic and patient characteristics. With this risk assessment model, patients at risk for CAI may be identified and properly informed on the treatment options. Patients identified as being at risk, may receive more adequate follow-up and become eligible for early surgical stabilization. This prevents patients from experiencing unnecessary long-lasting complaints, increasing the success rate of conservative and surgical treatment.

**Trial registration:**

Retrospectively registered:

NCT02955485 [Registration date: 3–11-2016].

NTR6139 [Registration date: 3–1-2017].

## Background

Chronic ankle instability (CAI) is a worldwide common sports orthopaedic problem. It mainly results from a lateral ankle sprain, progressing in up to 30–40% of patients to CAI despite adequate initial treatment [1, 2]. It is mainly defined as persisting complaints of giving way, pain and recurrent ankle sprains for at least 12 months [3]. Patients with CAI are generally eligible for surgery, whereas in the Netherlands in case of acute ankle instability mainly only professional athletes qualify for surgical stabilization [4, 5].

Repeatedly spraining the ankle, as occurs in patients with CAI, can have serious consequences such as damage to the ankle joint and osteoarthritis on the long term [6]. When early surgical treatment is performed, it leads to better results compared to late surgical repair after both ruptures and ligament laxity [7]. However, currently it cannot be adequately assessed which patients will not respond to conservative treatment and will develop CAI directly after an ankle sprain.

To determine who is eligible for early surgical treatment the contributing risk of prognostic factors should be determined. Many prognostic and risk factors for ankle sprains and ankle instability have been studied. Kobayashi et al. [8] wrote a critical review in 2014 outlining the many neuromuscular factors that have been studied and whether they are correlated to ankle instability. They concluded it was difficult to compare the findings as there was a lack of standardization for inclusion. Other factors that have been studied on their potential contribution to ankle instability are factors such as patient characteristics [9, 10], foot and ankle configuration [11–15], joint pathology [16–18] and treatment [19–23].

A large amount of factors associated with ankle sprains are of neuromuscular origin. An adequate rehabilitation program may help prevent progression to chronicity [8]. Other factors, for example patient characteristics, foot and ankle configuration and joint pathology, are already present at time of the initial sprain. These factors will not be positively influenced by a functional training program in contrast to neuromuscular associated factors. A risk assessment model based on factors that cannot be changed by rehabilitation programs, using standardized inclusion criteria, may therefore provide a reliable risk analysis on the development of chronic complaints.

### Aim of the study

To identify which patient characteristics and joint geometric factors are associated with development of CAI after an ankle sprain and subsequently develop and validate a risk assessment model to indicate which patients are at risk for developing CAI.

## Methods/Design

### Study design

This level II observational prospective cohort study, executed in 4 different Dutch hospitals, will include all subsequent patients visiting the Emergency Department (ER) reporting for a first-time sustained ankle inversion injury. These patients will be asked to fill out a questionnaire at 2 weeks, 3-, 6- and 12 months after their ankle sprain of which only the 12 month follow-up questionnaire is mandatory to diagnose CAI.

**Fig. 1:**
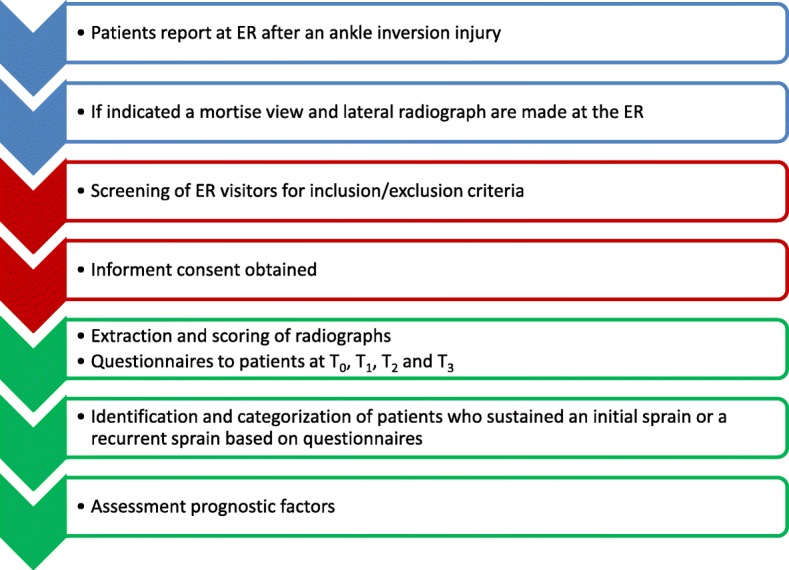
Study outline. Abbreviations: ER: Emergency Department; T_0_: 1st baseline questionnaire; T_1_: 2nd questionnaire at 3 months; T_2_: 3rd questionnaire at 6 months; T3: 4th questionnaire at 12 months. Blue: Standard care at the ER; Red: Inclusion phase; Green: Included patients

The study design (Fig. 1) is in accordance to the principles of the Declaration of Helsinki (version 10, October 2013 by 64th WMA General Assembly, Fortaleza, Brazil) and in accordance with the Medical Research Involving Human Subjects Act of the Netherlands. The study is approved by the local institutional review board (IRB) of the Academic Medical Center (AMC) and Flevoziekenhuis (W16_258 # 16.303), the Slotervaart Medical Center (U/16.130/P1654) and the VU Medical Center (VUmc) (2016.541).

### Study centres and participants

This study will be conducted at the ER of four Dutch hospitals: the AMC Amsterdam, Flevoziekenhuis Almere, Slotervaart Medical Center Amsterdam, VUmc Amsterdam. All subsequent patients that present themselves at the ER of one of the participating hospitals after an initial lateral ankle sprain will be treated according to standard protocol, including anamnesis, physical examination and (in case of positive Ottawa Ankle Rules (OAR)*) a radiograph [24]. When a mortise view and lateral radiograph is made and negative for ankle fractures or other joint pathology, patients are screened for eligibility according to the in -and exclusion criteria (Table 1).

**Table 1:** Inclusion- and exclusion criteria

Inclusion criteria	Exclusion criteria
At least 16 years old	Present fracture or other joint pathology/bone matrix pathology
Visited the ER within one week after a lateral ankle sprain	A diagnosed OCD after primary inclusion
Agreed with being approached for this study	Medial ankle instability
Reported anterolateral ankle pain after an ankle sprain or ankle distortion	Previous ankle surgery
Filled out the baseline, 3-, or 6 month questionnaire; and the 12 month follow-up	An unreliable radiograph due to the angle in which it is made or low quality
	Acute surgical repair or the ATFL/CFL or any other form of surgery within 12 months after the ER visit

*Abbreviations*: *ER* Emergency Department, *AP* anteroposterior, *OCD* osteochondral defect, *ATFL* anterior talofibular ligament, *CFL* calcaneofibular ligament

*The OAR are a requirement at the ER according to Dutch protocols to conclude whether there is an indication for a radiograph. However, in cases of severe LAS patients there is a high risk at false positive OAR despite the absence of a fracture (due to a painful lateral malleolus with or without the ability to bear weight).

### Definition CAI

Chronic ankle instability is defined as: *The perception of ‘giving-way’, in combination with a history of recurrent ankle sprains, the sensation of ankle instability and persistent disabilisty (pain, swelling* etc.*) that has not resolved in the time-frame from the first initial sprain up to the 12-months follow-up* [3, 25, 26]*.*

### Study procedure

#### Recruitment

All patients that sustained an (initial or recurrent) ankle sprain and of whom a mortise view and lateral radiograph is made, will be identified per ER of each participating hospital. These patients will be screened according to the in- and exclusion criteria (Table 1). All patients eligible for inclusion were approached for verbal informed consent. Patients are selected based on presentation at the ER. As these patients, out of the many patients who sustain an initial ankle sprain, experience severe enough complaints to actively reach out for help, these sprains may be regarded as ‘severe’ and therefore these patients may be more prone to persistent complaints. Also these patients can be monitored in case of a high risk of CAI in case of an initial sprain, as they have already been identified by the medical healthcare system, and radiographs have been made (according to standard protocol avoiding extra medical costs) which may be used to identify the size of the risk at CAI. All patients that were included and already suffered from recurrent ankle sprains, or CAI even, received the same follow-up as the patients who suffered from an initial sprain and were included in the sub-group analysis.

#### Follow-up

A legally required waiting period of two weeks is observed prior to asking consent and sending the questionnaires to the patient’s home address or email address. Patients are asked to fill out the first questionnaire immediately which gives a T0 (baseline measure at approximately two weeks after the initial sprain). The subsequent evaluation time points are T1 interim evaluation after three months, T2 after six months, and T3 at twelve months. The questionnaires contain questions about patient characteristics, previous ankle sprains, treatment, restrictions and complaints. Additionally the questionnaires contains the Foot and Ankle Outcome Score (FAOS) and the Cumberland Ankle Instability Tool (CAIT) to evaluate recovery. To ensure all patients with CAI are identified, one year after their ER visit they will be contacted, inquiring on persisting complaints and ankle instability. Patients that indicate on the first questionnaire they sustained ankle sprains prior to their ER visit will not receive further follow-up questionnaires, as these patients are considered to have CAI and therefore need no extra monitoring to identify whether they develop CAI or not. They will not be included in the primary analysis as this may lead to overfitting of the current model. Concomitant neuromuscular adaptations and additionally may follow a different rehabilitation route due to familiarity with the complaints.

In case of persisting complaints six months after the ER visit, patients will be eligible for surgical treatment according to Dutch standards. This is to avoid unnecessary invasive treatment on patients as a large proportion recover by means of conservative treatment [7].

### Clinical outcome measures

**Fig. 2:**
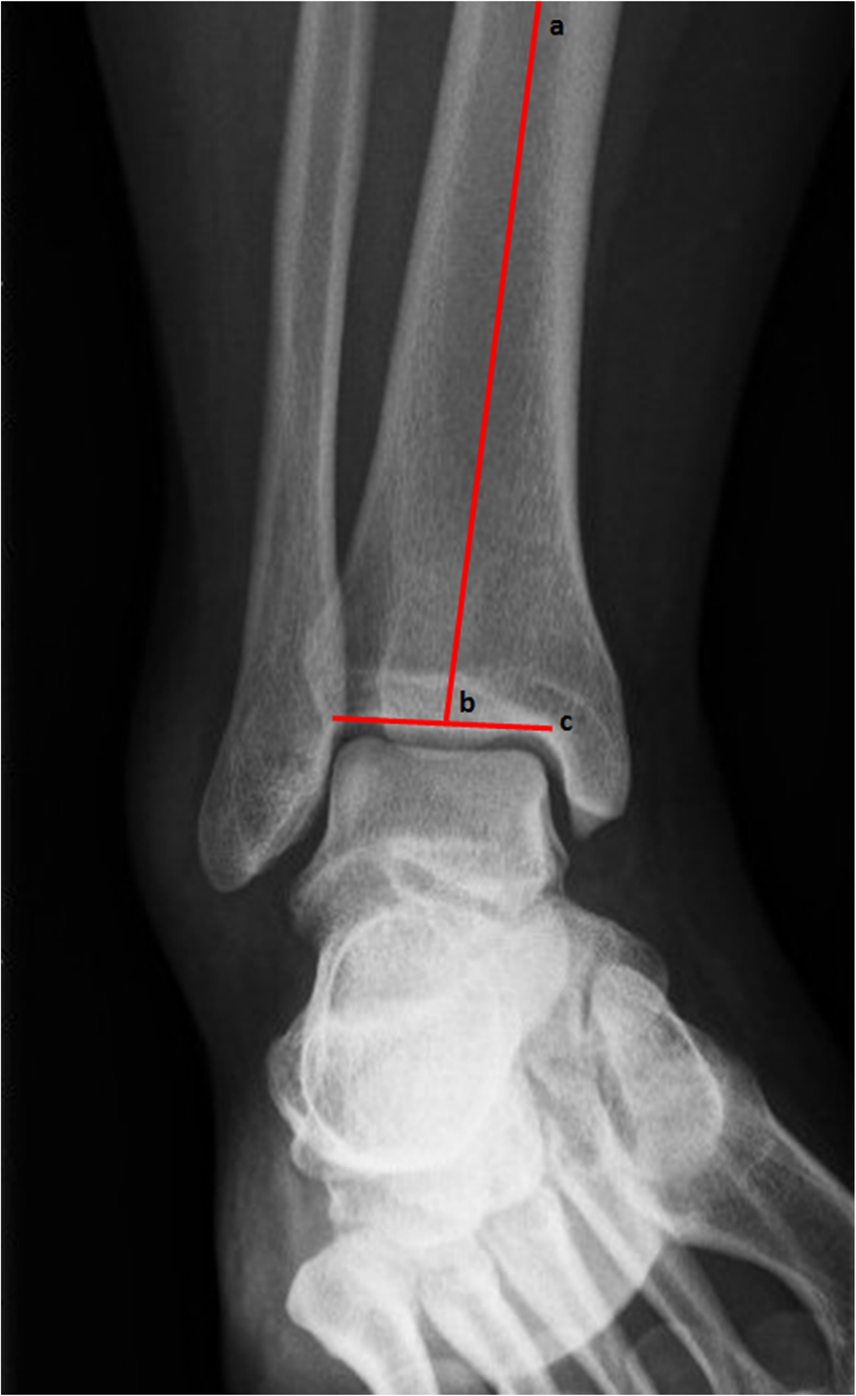
Alignment assessment using the medial distal tibial angle (**b**). This angle (arrows) is formed by the tibial axis (**a**-**b**) and the joint orientation line of the distal tibia (**c**). The MDTA was measured in two ways: (1) the absolute angle and whether this angle included a (2) varus (<87^o^), normal (87-91^o^) or valgus (>91^o^)

**Fig. 3:**
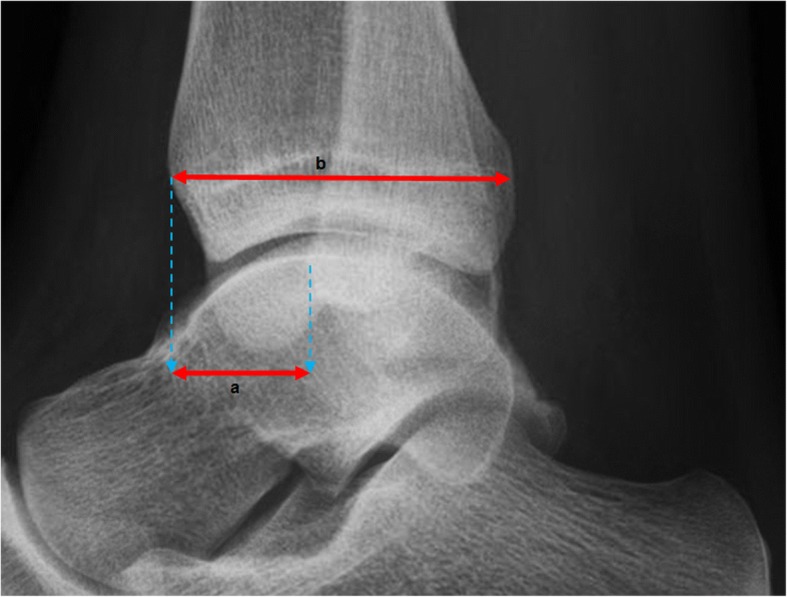
The fibular position is assessed drawing two lines from the edge of the anterior tibia to the posterior part of the tibia (**b**) cq. the anterior part of the fibula (**a**). The absolute distance is measured using the projected posterior distance in millimeters (mm) of the fibula in relation to the tibia (height a in mm); and the relative fibular position is assessed as a posterior position in terms of percentage (a divided by b in %)

**Fig. 4:**
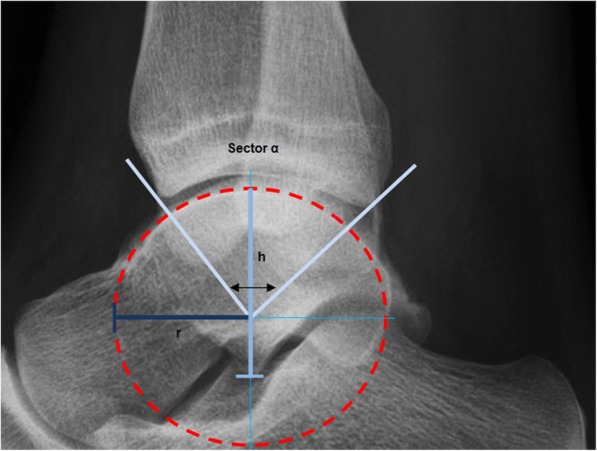
The tibiotalar contact ratio is assessed using 3 variables: the talar radius (**r**); the talar height (**h**); and the tibiotalar sector (**α**). By drawing a circle digitally fitted to the talar joint surface, the radius is determined by drawing a line to the middle of the circle, measured in millimeters (**r**). The height is determined drawing a line through the middle of the circle from the surface of the talus to the inferior border, measured in millimeters (**h**). From the center of the circle two lines are drawn to the anterior and posterior margins of the distal tibia, representing the tibiotalar sector (**α**)

**Fig. 5:**
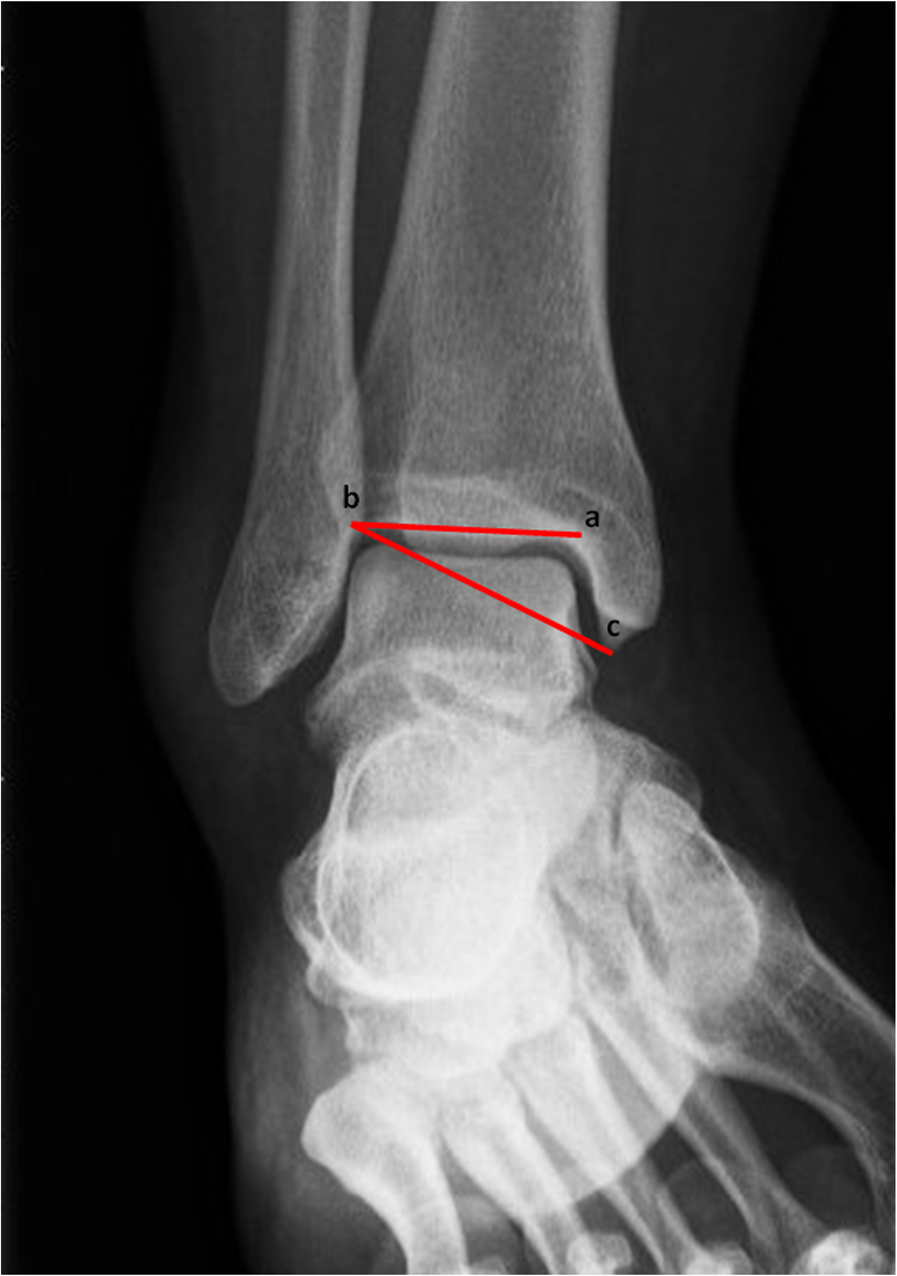
Medial Malleolar Height Angle (MMHA) formed by the angle connecting the distal tibial joint line (**a**-**b**) and the line from the most lateral point of the tibiotalar joint line to the most distal point of the medial malleolus (**b**-**c**)

**Fig. 6:**
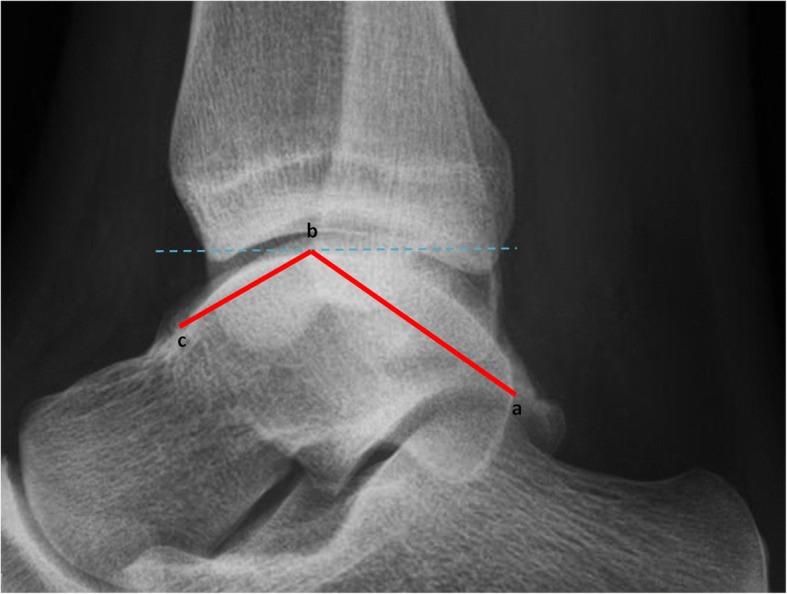
The Talar Convexity Angle (TCA) is formed by connecting the posterior talar tubercle (**a**), the most proximal part of the talus (**b**), and the deepest point of the transition to the talar neck (**c**)

The first outcome measure is the radiograph made at the ER. This radiograph will be assessed for nine bone geometric characteristics. These characteristics will be implemented in the model in case they show a correlation with developing CAI: ankle alignment using the medial distal tibial angle (MDTA) measured as (1) the absolute angle and whether this angle was considered a (2) varus (<87^o^), normal (87-91^o^) or valgus (>91^o^) position [27], fibular position in relation to the tibia measured as (3) projected distance and (4) relative distance [28], and tibiotalar contact ratio assessing the (5) talar height, (6) radius and (7) angle between the talus and tibia, (8) the angle representing the height of the medial malleolus and (9) the talar curvature (Figs. 2, 3 and 4) [29]. Additionally (10) the Medial Malleolar Height Angle (MMHA) and (11) the Talar Convexity Angle (TCA) [30] (Figs. 5 and 6).

Secondly, (12) age, (13) sex, (14) height, (15) BMI and (16) sports intensity (by means of the Ankle Activity Index (AAI)) will be implemented in the model as important patient characteristics, in case of a significant correlation. All these variables have shown a (prospective or retrospective) correlation with ankle instability in previous research apart from MDTA, MMHA and TCA. These three, however, have shown a relation with other pathologies that also concern an inversion-plantarflexion trauma mechanism and will therefore be explored on a potential correlation with CAI [14, 16, 30].

To assess overall recovery and CAI at 12 months, a questionnaire including patient reported outcome measures (PROMs) will be used. This questionnaire includes the FAOS and CAIT. The FAOS is a Dutch validated questionnaire assessing pain, other symptoms (swelling, locking, mobility, stiffness), function (activities of daily living) and sports and foot- and ankle related quality of life divided over 5 subscales, with scores ranging from 0 to 100 [31, 32]. The CAIT is scale consisting of 9-items measuring the severity of functional ankle instability per ankle individually. The total score ranges from 0 to 30 with a cut-off value discriminating between a functionally stable and instable ankle of 11.5 points (as calculated for the Dutch population) [33, 34].

### Analyses

#### Inter- and intra-observer reliability

Two radiology residents, an orthopedic resident and an emergency physician of the AMC will be included in the inter- and intra-observer reliability assessment. These professionals are selected because the orthopedic surgeon eventually treats the patient with CAI, and the emergency physician is the first to see a patient at the ER after a severe ankle sprain and the radiologists assess the radiographs. Radiologists are most experienced in radiographic assessment and are therefore be considered as reference standard, i.e. to assess inter-observer reliability the measurements by the orthopedic resident and emergency physician are compared to the measurements performed by the radiology residents. To assess intra-observer reliability 39 radiographs are assessed twice by each observer with a minimum of 2 weeks in between. In order to prepare the reliability assessment a calibration session will be held with all observers. If this proves the measurements can be performed reliably they will be implemented in the other participating hospitals after a calibration meeting and practice session.

#### Prognostic value of variables

To determine whether the variables, selected from current literature, can be implemented in our model, it has to be determined whether they are of prognostic value on the progression of an ankle sprain to CAI. This will be done by determining whether they have an individual correlation with instability 12 months after an ankle sprain and whether this correlation is influenced using a multivariate analysis. In case of no existing correlation, factors are left out of the model and the sample size is recalculated accordingly.

#### Prognostic model

The main objective of this study is to assemble the required data and subsequently construct a risk assessment model for the development of CAI after a first initial ankle sprain based on patient characteristics and the ankle joint geometric characteristics. This model is based on fourteen factors that are assessed using a mortise view and lateral radiographs and a questionnaire. The patient characteristics that are implemented in the model are age, sex, height, BMI and sports intensity. Even though BMI and sports intensity may be considered as modifiable, BMI reflects the current situation and sports intensity is the level a patient wishes to return to, additionally reflecting the risk at ankle sprain exposure. Additionally nine bone geometric characteristics are implemented in the model (See clinical outcome measures).

#### Additional analyses

By comparing PROM scores over time, overall recovery will be assessed. In addition, the different treatments and symptoms during physical examination will be assessed for their relation to the functional outcome, as measured by the FAOS and CAIT and persisting complaints of instability. The final sub-analysis differentiates between the amount of recurrent ankle sprains prior to the initial ER visit to check whether the model is also applicable on patients that already suffer from CAI, as identified by the first questionnaire.

### Sample size calculation

#### Reliability assessment

For the inter- and intra-observer reliability with an expected agreement of 80% with a relative error of 20%, a sample size of 39 patients is calculated. This sample of 39 will be a subsample from the cohort of the risk assessment model [35].

#### Assessment of agreement

Assessment of the prognostic value of the fourteen variables will be performed on the sample size of the internal validation [36]. To avoid overfitting, the prognostic value of these 16 variables will be assessed when 10 events per variable (EPV) is reached [37]. With a maximum of sixteen variables with a minimum of 10 events per variable, a prevalence of 30–40% after an initial sprain, including a 10% loss-to-follow-up, a sample size of 593 patients is needed to assess their prognostic value (Table 2).

**Table 2:** Sample Size calculation prognostic value, internal validation [38, 39]

EPV = no. of events/no. of regression coefficients		
Minimal EPV	≥10 Prognostic Value	≥20 Prediction Model
Change of developing CAI	30–40%	30–40%
No. of regression coefficients	16	16
(? = no of events needed)	10 =?/16 ? = 160 minimal 160 events	20 =?/16 ? = 320 → minimal 320 events
In case of 30% chance of developing CAI	160*3.33 = 532.8 patients	320*3.33 = 1065.6 patients
In case of 40% chance of developing CAI	160*2.5 = 400 patients	320*2.5 = 800 patients
Sample Size (incl. 10% loss to follow-up)	445–593 patients	889–1185 patients

#### PREDICT sample size

For a prognostic model at least 20 EPV is needed to optimize reliability and to avoid bias in regression coefficients [37–39]. Based on having maximally 16 variables with a minimum of 20 events per variable, a prevalence of 30–40% after an initial sprain, including a 10% loss-to-follow-up, a sample size of 1185 patients is needed to perform the internal and 1185 patients is needed to perform the external model validation, resulting in a total of 2370 patients (Table 2).

During the study the required sample size may change and will be recalculated accordingly in case one of the following scenarios occur:

- In case one or more radiographic factors have a low inter- or intra-observer reliability (ICC ≤0.70), they will be excluded from the risk assessment model and the sample size will be recalculated using the new number of prognostic factors. If radiographic assessments cannot be performed in more than 2 cases (> 5%) a candidate predictor will be excluded, as the problem is likely to recur and therefore cannot be adequately assessed, now and during future assessment (e.g. up to 5% missing data is accepted) [40, 41].
- In case of multicollinearity between (ICC > 0.70) between prognostic variables one will be excluded;
- If a variable lacks agreement (ICC ≤0.70) with CAI it will be excluded;
- In case the prevalence of CAI during the study differs from the estimated 30%. In case the percentage of patients progressing to CAI exceeds the 50%, the sample size calculation will be based on the number of non-events. The prevalence will be recalculated when 500, 750, 1000, 1250, 1500, 1750,2000 and 2250 patients have returned the last questionnaire to minimize patient burden, as inclusion will stop as soon as the minimal required sample size is reached.

### Data analysis and statistics

Demographics of the study population will be analyzed using the descriptive statistics. All statistics will be performed with SPSS version 23.0 (SPSS Inc., Chicago, IL, USA) and R package ‘Stats’ version 3.3.3 (University of Auckland, Auckland, New Zealand). A *p*-value of < 0.05 is considered significant. Normality of data will be assessed using the Shapiro-Wilks test.

#### Inter- and intra-observer reliability

The inter- and intra-observer reliability will be analyzed using the Intraclass Correlation Coefficient (ICC) Two-Way Mixed model on absolute agreement in case of normally distributed data. Reliability is considered poor if the ICC is ≤0.40, moderate between 0.40 and 0.70, good between 0.70 and 0.90, and excellent > 0.90 [41]. In case of skewed data the Cronbach’s alpha will be calculated. In case of an ICC of > 0.70 a Bland and Altman plot will be drawn to visualize the measurements and their relation to the limits of agreement.

Additionally the Standard Error of Measurement (SEM: SD x √(1-ICC)) and Minimum Detectable Change (MDC_95%_: 1.96 x √2 x SEM) \ will be calculated to objectify the ICC.

#### Prognostic value of variables

Depending on normality of data the Pearson or Spearman correlation coefficients will be used to assess correlations. A scatterplot for numerical data and Odds Ratio (OR) for binary data will be used to provide additional information on possible correlations in the data between prognostic factors. In case of correlations (*p* < 0.10 to avoid overfitting [40]) shown by an univariate analysis, a multivariate logistic regression will be performed to assess dependency between variables. The R^2^ will be used to visualize model fitness for the contribution of a factor to the progression to CAI and for the full model.

#### Prognostic model

For internal validation a maximal amount of data is required to increase the reliability, therefore apparent validation will be used, including the full sample size of 1026 patients. To increase reliability the bootstrapping procedure will be used in the development of the model. A sample of 1000 bootstraps will be used to assess bias in the regression coefficients. Additionally the sensitivity, specificity and ROC curve will be used to assess the predictive value of the model.

To define generalizability of the created model, temporal validation is chosen to decide whether patients will be included in the internal or external validation database. After determining the prognostic value of the included variables, the full required sample size is calculated. Additionally the duration until this sample size will be reached, is estimated. Subsequently the time stamp for the temporal validation is decided. This ensures both sample sizes will be sufficient and reached. Again the sensitivity, specificity and ROC curve will be used to assess the suitability of the model [36].

Variables with a significant correlation with CAI (age, sex, height, BMI, sports intensity, varus/valgus alignment, fibular position, tibiotalar contact ratio, height medial malleolus and talar convexity), in addition to a continuous model, will be categorized to provide a binary model that can be easily implemented in daily practice by simply answering question by ‘yes’ or ‘no’. For this the Youden index and a ROC curve will be used [33]. For both models the final objective is to create a model with cut-off values that advises surgery, watchful waiting or no surgical treatment by combining the scores with the patients expectations in the shared decision making process.

The cut-off values will be reflected to the MDC, SEM and MCID to ensure minimal measurement bias.

#### Additional analyses

To assess overall recovery, mean and Standard Deviation (SD) of the functional outcome scores are calculated using the paired T-test. In case of skewed data the Wilcoxon signed rank test will be used instead, reporting the median and range.

To assess whether the received treatment affects functional outcome and/or the progression to CAI will be assessed. Depending on normality of data the Pearson’s or Spearman’s rho will be used.

Finally based on the amount of ankle sprains experienced prior to participation in this study, patients will be defined as suffering from an acute lateral ankle sprain (LAS) or CAI. The correlation coefficient of acute LAS and bone geometric factors, and CAI and bone geometric factors will be calculated using the Pearson’s or Spearman’s rho (depending on normality or skewed data). These correlation coefficients will be compared to see whether there is a difference in bone geometry between acute LAS or CAI patients.

### Missing data

A per protocol analysis is applied based on patients filling out at least one questionnaire and having an available AP and lateral radiograph for radiographic assessment. In case the last questionnaire is missing, on which the diagnosis of CAI will be based, and the question concerning persisting complaints at six or twelve months is not answered, differentiation between full recovery or progression to chronicity cannot be made. For this reason these patients and patients with a missing radiograph will be excluded.

### Adverse events

No study-related adverse events are expected as the study contains an observational cohort with 1 set of radiographs made according to standard protocol.

## Discussion

Currently patients with CAI suffer for an unnecessary long period of time as they are unaware of treatment option and often are not eligible for direct surgical stabilization. By developing a risk assessment model tested for its validity, patients at risk may be identified and receive appropriate treatment. The current model is the first to address the progression to CAI on a large scale including both internal and external validation. Focusing on bone geometry, sex and height means focusing on factors patients cannot control despite their potential role in progressing to chronicity [8]. As patients will want to return to their old level of sports, the additional inclusion of sports intensity reflects the risk of exposure to loading events that cause recurrent sprains. BMI and height, despite their strong correlation, are included as they both have been extensively described as a prognostic factor and should be included. However, as the inclusion of both factors will create bias, the factor with the strongest correlation with CAI will be included in the model.

In case of CAI, patients become eligible for surgical stabilization after 6 months of failed conservative treatment [4, 5]. Normally after an ankle sprain, it is assumed that the complaints will automatically resolve, and physicians lose track of the patients and thus do not have a proper follow-up. Patients that progress to CAI, unaware of treatment options, often experience complaints for unnecessary long period of time.

With a risk assessment model that has the potential to identify patients at risk for CAI, patients can be properly informed on treatment options prior to becoming chronic. Patients pre-identified as being at risk, may receive adequate follow-up [42]. Additionally knowing about the potential course of complaints and treatment options, patients that still experience complaints after 6 months, and therefore progressed to chronic instability, can ensure they are seen by a physician on time. This way they can become eligible for relatively early surgical stabilization, not experiencing complaints for an unnecessary long period of time, in turn increasing the success rate of surgery [7].

By including only patients that visit the ER and of whom a radiograph is made due to positive OAR, only the ‘severe’ ankle sprains are included. For these patients persisting complaints are most expected. Additionally, these patients remain in the picture by the treating physicians and can therefore receive proper follow-up. However, the strictness of following the OAR per ER is unknown and this creates a selection bias because not all sprains, of which a radiograph is made, score positive on the OAR. Also patients that were already experiencing chronic complaints may be included. For this reason an extra analysis will be performed to assess how many patients already experience recurrent sprains and whether the model is also fitting for these patients. However, it can be argued that, if the prognostic model fits the patients that develop CAI, it should also fit the patients that have already progressed to CAI. Another possible limitation of this study is the unknown adequacy of the specific set of selected radiographic factors that will be implemented in this model. By means of a literature search multiple radiographic characteristics that may be correlated to CAI were identified. However, most were validated using weight-bearing radiographs, which cannot be made after sustaining a severe ankle sprain. For this reason, the subtalar and talar tilt angle are excluded from this study as these are dependent on a weight-bearing condition.

There is a rising demand for prognostic models that aid in risk analysis [43–46] and choice of treatment [44, 47–49]. Most models reflect the need of large study cohorts and multiple measurements over time [43, 47, 49]. However, sample size calculation and power analysis are often absent [44, 46, 49–51] or studies include a convenience sample based on the amount of retrospective data available [43, 45, 47]. This often leads to an imbalance between the number of studied predictive factors and the number of patients with the studied condition. Despite the claim of (internal and/or external) validity of the model, methods for validation are not described [49] or are absent [50]. To ensure we would not encounter these problems, our sample size and methods are developed following state-of-the-art directions [36]. Doing so, with a relatively heterogenic population, we anticipate to create a valid risk assessment model, based on easily identifiable factors and well suited for severe ankle sprain patients in clinical settings outside the current study settings. A valid risk assessment model may facilitate clinical decision making when a low or high risk at a certain disorder has been identified. This article may function as State-of-the-Art support for other researchers, interested in prognostic factors and in developing their own model to improve clinical practice.

## Abbreviations

AAI: Ankle activity index
AMC: Academic Medical Center
BMI: Body Mass Index
CAI: Chronic ankle instability
CAIT: Cumberland ankle instability tool
EPV: Events per variable
ER: Emergency department
FAOS: Foot and ankle outcome score
ICC: Intraclass correlation coefficient
IRB: Institutional review board
LAS: Lateral ankle sprain
MDTA: Medial distal tibial angle
MM: Millimeters
OAR: Ottawa ankle rules
PROM: Patient reported outcome measure
SD: Standard deviation
SDC: Smallest detectable change
SEM: Smallest detectable change
T0: First questionnaire two weeks after consent is received
T1: Second questionnaire after 3 months
T2: Third questionnaire after 6 months
VUmc: VU Medisch Centrum
